# Multimodal framework for swallow detection in video-fluoroscopic swallow studies using manometric pressure distributions from dysphagic patients

**DOI:** 10.1007/s11548-025-03556-1

**Published:** 2025-12-15

**Authors:** Manuel Maria Loureiro da Rocha, Lisette van der Molen, Marise Neijman, Marteen J. A. van Alphen, Michiel M. W. M. van den Brekel, Françoise J. Siepel

**Affiliations:** 1 Robotics and Mechatronics Group, University of Twente, Drienerlolaan 5, Enschede, 7522 NB the Netherlands; 2 Head and Neck Oncology, Netherlands Cancer Institute, Plesmanlaan 121, Amsterdam, 1066 CX the Netherlands

**Keywords:** Head and neck cancer, Impedance manometry, Video fluoroscopy, Automatic swallow detection, Pressure template

## Abstract

**Purpose:**

Oropharyngeal dysphagia affects up to half of head and neck cancer (HNC) patients. Multi-swallow video-fluoroscopic swallow studies (VFSS) combined with high-resolution impedance manometry (HRIM) offer a comprehensive assessment of swallowing function. However, their use in HNC populations is limited by high clinical workload and complexity of data collection and analysis with existing software.

**Methods:**

To address the data collection challenge, we propose a framework for automatic swallow detection in simultaneous VFSS-HRIM examinations. The framework identifies candidate swallow intervals in continuous VFSS videos using an optimized double-sweep optical flow algorithm. Each candidate interval is then classified using a pressure-based swallow template derived from three annotated samples, leveraging features such as normalized peak-to-peak amplitude, mean, and standard deviation from upper esophageal sphincter sensors.

**Results:**

The methodology was evaluated on 97 swallows from twelve post-head and neck cancer patients. The detection pipeline achieved 95% Recall and 92% F1-score. Importantly, the number of required HRIM annotations was reduced by 63%, substantially decreasing clinician workload while maintaining high accuracy.

**Conclusion:**

This framework overcomes limitations of current software for simultaneous VFSS-HRIM collection by enabling high-accuracy, low-input swallow detection in HNC patients. Validated on a heterogeneous patient cohort, it initiates the groundwork for scalable, objective, and multimodal swallowing assessment.

## Introduction

### Head and neck cancer and oropharyngeal dysphagia

The swallowing mechanism is responsible for the adequate delivery of nutrients and water, necessary for the normal function of our body. This physiological process entails a sequence of coordinated muscle contractions and distensions, subdivided into three anatomical phases: oral, pharyngeal, and esophageal [1].

Dysphagia is defined as a disturbance or impairment of the swallow mechanism resulting from structural, neurological, or functional impairments [2, 3]. Patients diagnosed with head and neck cancer (HNC), i.e., malignancies of the oral cavity, pharynx, or larynx, are very likely to develop oropharyngeal dysphagia (OD) due to functional impairments and/or anatomical changes post-treatment [4]. OD often leads to dehydration, malnutrition, aspiration-induced pneumonia, airway obstruction, and even death [2, 3]. Given the high incidence and complications of OD in the HNC population, the clinical focus is not only on achieving an accurate diagnosis but also on the development of more personalized and effective treatment plans [5, 6]. To this end, the accurate quantification of the swallowing function could serve as a valuable tool [6].

Video-fluoroscopic swallow study (VFSS) is one of the standard diagnosis tools of OD [7]. During VFSS, patients swallow boluses of different volumes and consistencies, mixed with a contrast agent, while a fluoroscopy video of each single-bolus swallow is recorded [8]. From these videos, it is possible to characterize the swallowing process and identify specific dysfunctions [9]. Nevertheless, expertise and specialized training are needed for an accurate diagnosis, specifically in HNC patients, making it prone to human errors and inter-rater variability [10, 11].

**Fig. 1:**
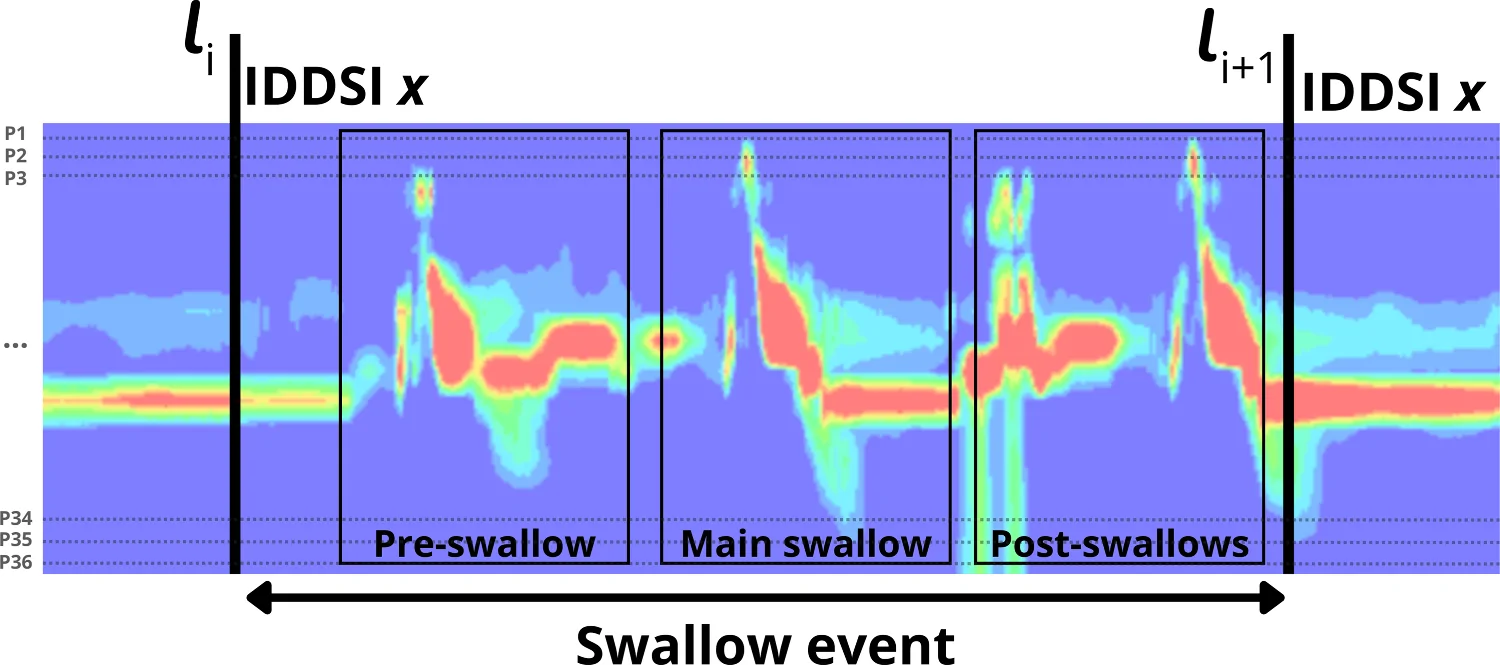
Example of HRIM pressure contour plot with onset and offset landmarks, $l_i$ and $l_{i+1}$, respectively, and pressure sensors (P1 to P36) displayed along the vertical axis. The region between the landmarks is a complete swallow event (Definition 1) with label IDDSI*x*, indicating the type of swallow based on viscosity and volume, according to the International Dysphagia Diet Standardisation Initiative (IDDSI) [16]. Within a swallow event it is possible to find multiple swallow attempts, namely *Pre-* and *Post-swallows*, as well as the *Main swallow*. This example is merely illustrative and does not represent a real case

High-resolution impedance manometry (HRIM) is a validated tool, composed of a sequence of pressure and impedance sensors, that offers valuable quantitative measurements of the swallowing function by assessing swallowing pressure dynamics and bolus flow resistance [12, 13]. The analysis of HRIM is particularly complex and demanding in patients with altered anatomy, such as HNC patients, where reduced pharyngeal pressures are frequently observed, and multiple swallows are required to clear the bolus (cf. Fig. 1) [14, 15]. Additionally, in HRIM measurements the analysis depends on the clincian’s expertise, which can often lead to a less accurate dysphagia diagnosis and swallowing quality assessment, potentially resulting in the development of a suboptimal treatment plan.

Combining HRIM with a gold-standard diagnostic tool like VFSS offers clinicians complementary information that enhances the objectivity, reliability, and completeness of OD assessment, particularly in the HNC population [6, 17, 18]. On the other hand, the analysis process becomes significantly more complex and time-consuming as clinicians must perform the standard analysis of both data sources [12].

Semi-automated analysis software such as ManoScan$^{TM}$ (Medtronic®) and Solar GI$^{TM}$ (Laborie®) enable synchronization of HRIM and VFSS from the time of acquisition. However, these systems typically record VFSS segments within fixed time windows following the placement of the swallow onset marker, which introduces two major limitations. First, this approach fails to accommodate the variability of swallow events (cf. Definition 1) characteristic of the HNC population. More importantly, it increases the clinical workload by requiring clinicians to manually annotate HRIM data and supervise the VFSS acquisition process, diverting attention from patient care and introducing potential inconsistencies in synchronization.

### Our contribution

In this paper, we introduce a novel autonomous framework for localizing swallow events in VFSS recordings from simultaneous multi-swallow VFSS and HRIM collections. The main contributions are:

1. *Robust and cross-verified swallow detection:* Swallow candidates (Definition 2) are detected in VFSS videos using an optimized double-sweep OF algorithm. A pressure-based swallow template is constructed from pre-annotated HRIM swallows and used to classify the swallow candidates. This enables a two-way verification strategy that not only isolates swallows present in the VFSS stream, as it recovers relevant manometric data that may have been missed during the live HRIM annotations.
2. *Reduced clinical workload and manual input:* By automating the detection and classification process, the framework significantly reduces the workload of clinicians in assessing swallowing function in HNC patients. This approach represents an important step toward fully automating diagnostic examinations, ultimately eliminating the need for manual input and enhancing consistency in swallow assessment.

The proposed framework was validated using data from HNC patients, a group characterized by a high incidence of swallow abnormalities, where standard VFSS and HRIM assessments are prone to errors that can impact patient outcomes.

### Related work

Motion detection is a widely explored area in computer-assisted medical imaging. A commonly used technique for this task is optical flow (OF). Among various OF methods, the Farnebäck dense optical flow algorithm [19] is one of the most widely adopted due to its robustness across different imaging modalities and tasks. In fluoroscopy, for example, it has been recently used to estimate bowel sounds [20], assess chest motion [21], and segment lung nodules [22].

VFSS analysis over the last decades has focused on automating and enhancing the diagnosis of OD by aiding human analysis. Specifically, deep learning approaches have increasingly supplanted semi-automated methods to handle complex tasks and streamline the diagnostic workflow [23]. These models have been used to detect and track key anatomical landmarks [24, 25], track the bolus [26–28], identify the pharyngeal phase of swallowing [28, 28, 29], and detect penetration or aspiration events[30–34]. Despite their versatility and accuracy, deep learning models, as opposed to knowledge-based methods, require large well-annotated datasets to achieve reliable and generalizable results, as well as high-computational resources for real-time applicability [23].

A particular study by Lee et al. [35] combined Total Variation–L1 norm (TV-L1) OF with deep learning networks to detect the pharyngeal phase in VFSS video segments. Their dataset included non-swallow events, such as coughing and camera adjustments, which the authors cite as the motivation for employing a large 3D deep learning architecture. To train this architecture, they required 3,354 annotated VFSS clips. When using TV-L1 OF flow fields alone, the presence of non-swallow events resulted in a high false positive rate of 50%. Similarly, Abdelaziz et al. [36] presented a toolkit to automate VFSS analysis. A key contribution of this work was the integration of various OF methods with deep learning architectures to detect individual swallow segments within VFSS data streams that also included swallow-unrelated movements. Despite achieving strong results, the authors relied on a large annotated dataset comprising 229,266 frames. Optimizing both approaches with a lightweight swallow detector with minimal data requirements could reduce their data burden and facilitate clinical applicability.

Since HRIM started showing its clinical value, multiple approaches attempted to tackle its disadvantages. Pioneer studies presented semi-automated approaches based on analytical methods, to detect swallow patterns and extract swallow quality metrics from healthy subjects [37, 38] and in dysphagic populations [39, 40]. Recent approaches aimed to detect and classify swallows on esophageal HRIM [41–43]. Despite the promising results of these approaches, their applicability to oropharyngeal HRIM and to clinical practice requires further studies due to insufficient reliability in patient populations with altered anatomies, such as HNC patients [44].

The combination of HRIM and VFSS has been adopted in clinical settings and has demonstrated improved accuracy and reliability in diagnosing OD [6, 18, 45, 46], particularly in the HNC population [15]. Nevertheless, despite its anticipated clinical benefits, current limitations in standardization and equipment hinder its adoption in the HNC population [15]. Moreover, the use of two diagnostic tools increases the clinical burden, and only a limited number studies have addressed workload reduction through automatic, computer-assisted methodologies. Studies by Jell et al. [47] and Geiger et al. [48] focused on integrating HRIM and VFSS data in a single user-interface based on time-synchronized signals. However, both approaches relied on the prior selection of swallow events, likely based on HRIM annotations. While the clinical feedback was positive, this reliance means that any unannotated swallow event in the HRIM data would be excluded from the analysis.

## Methodology

The proposed framework aimed to automatically detect single-bolus swallow events in multi-swallow simultaneous HRIM and VFSS, through VFSS OF analysis and HRIM pressure distribution categorization.

### Data collection protocol

The capture of simultaneous VFSS and HRIM was conducted by a trained speech language pathologists at the Netherlands Cancer Institute (NKI) (Amsterdam, the Netherlands). Fluoroscopy images were acquired at 25 frames-per-second using CombiDiagnost R90$^{TM}$ (Philips®, Amsterdam, the Netherlands), with continuous session recording via Bandicam$^{TM}$ (Bandicam Company®, Irvine, CA, USA) at 30 frames-per-second. The standard protocol of capturing fluoroscopic images only during active X-ray exposure was modified to allow continuous recording from the start of the examination. This adaptation addressed equipment limitations, particularly software constraints of the HRIM system, which can result in the loss of critical parts of the swallow event in the HNC population. Additionally, this adjustment allows a comprehensive retrospective analysis, including access to all verbal clinician comments. This recording is the main input of our methodology.

As in standard HRIM data collection protocols, single-bolus swallows were manually annotated by the responsible clinician during acquisition. At the NKI, both the onset and offset of each swallow event were marked directly on the HRIM stream, ensuring that the entire event was enclosed within these annotations (cf. Fig. 1). Similarly, each swallow event was guaranteed to be fully captured under X-ray. However, because HRIM landmark placement was independent of fluoroscopic swallows, the annotated HRIM segment and the corresponding VFSS video segment may differ in duration, despite capturing the same swallow event.

The solid-state manometer Solar GI$^{TM}$ K103659-E-1180-D (Laborie®, Portsmouth, NH, USA) recorded pressure and impedance data at 20 Hz. The manometer was equipped with 36 pressure sensors and 16 impedance sensors. Following Omari et al. [12] the HRIM catheter was inserted nasally to a depth of approximately 40 cm, until it cleared the UES and all pressure sensors were inside the patient. The simultaneous VFSS-HRIM protocol, adapted from Palmer et al. [8] and Omari et al. [12], involved lateral-view video recordings of patients swallowing boluses of varying consistencies and volumes, following the International Dysphagia Diet Standardisation Initiative (IDDSI) guidelines [16].

**Fig. 2:**
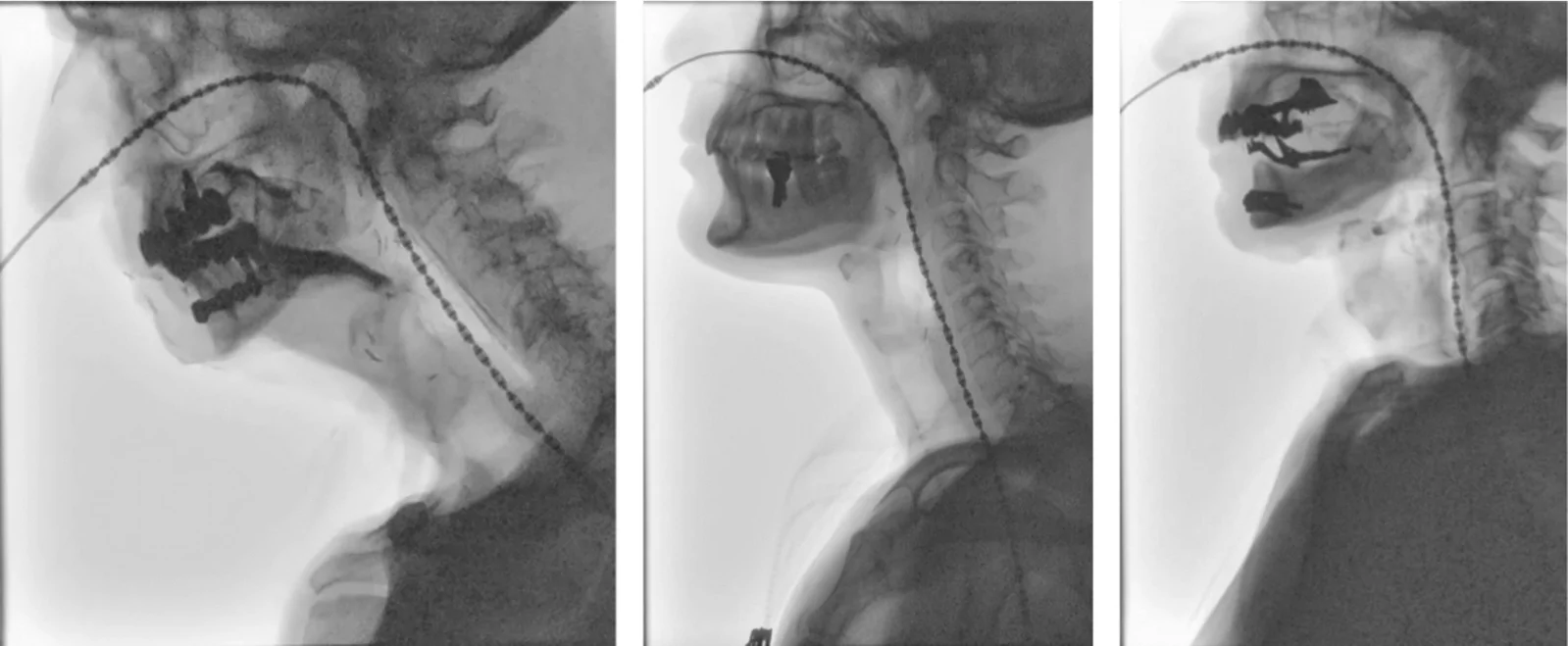
VFSS video frames representative of the standard positioning of patients during simultaneous HRIM and VFSS. All anatomical structures of upper aerodigestive tract are visible, as well as all the manometric sensors in this region

The study included 12 male post-HNC patients (mean age 66±11 years). Primary tumor locations were the oral cavity and the oropharynx, and the main treatment modalities were surgery and adjuvant radiotherapy. A complete cohort characterization is provided Table 3. The protocol aimed to collect at least one, and preferably two, samples per patient across two amounts (5cc and 10cc), and three consistencies: thin or slightly thin liquids (IDDSI 0–1), thick or extremely thick liquids (IDDSI 3–4), and solids (IDDSI 7). The protocol was adjusted as needed when patients were unable to safely swallow the required consistencies. For clinical analysis purposes, some patients were also asked to perform dry swallows. Patients were positioned to ensure visibility of all HRIM sensors, lips, oropharyngeal structures, vocal cords, and cervical vertebrae in the fluoroscopy images (cf. Fig. 2).

### Datasets

Simultaneous VFSS and HRIM data from 12 post-HNC patients were collected under the approval of the NKI-AVL Institutional Review Board (IRBd21–210, IRBd23–322).

Bandicam$^{TM}$ session recordings had an average length of 14.06±4.35 min, with an average exposure time of 3–4 min. An expert manually annotated each recording, marking the onset (start of irradiation) and offset (end of irradiation) of movement segments in the VFSS videos, and classified them as swallow or non-swallow. The dataset included 154 video segments: 97 swallow events, with an average length of 15.74±4.76 s; and 57 non-swallow segments. Furthermore, all 97 swallow events were annotated in the HRIM feed; however, 61 of these were intentionally treated as unannotated to enable validation of the algorithm’s accuracy and clinical relevance. Swallow consistencies (IDDSI levels) were extracted from session reports and linked to their corresponding video segments. This process resulted in a total of 22 *IDDSI 0* swallows, 15 *IDDSI 1* swallows, 3 *IDDSI 2* swallows, 14 *IDDSI 3* swallows, 16 *IDDSI 4* swallows, 1 *IDDSI 5* swallow, 18 *IDDSI 7* swallows, and 8 dry swallows.

The true delays between the Bandicam$^{TM}$ recording and the HRIM stream, $\Delta _{t_{\text {ref}}}$, were calculated using a synchronization procedure analogous to the one described in Sect. 2.4, using binary signals generated from the ground-truth (GT) swallow timestamps in each data stream. The reference delay was then defined as the time shift corresponding to the maximum cross-correlation between these two binary signals, under the condition that, after synchronization, the timestamps in both data streams were aligned such that the overlapping swallow labels (i.e., IDDSI levels) corresponded to the same swallow event.

### Automatic movement detection in continuous VFSS recordings

Accurate detection of movement segments in VFSS videos is essential for reliable swallow classification. To this end, we proposed an optimized double-sweep OF algorithm based on the Farnebäck dense OF algorithm [19].

#### Fast optical flow sweep

The *Fast OF sweep* provided a rough estimate of the video dynamics by sampling frame pairs at a sampling rate, $\Delta _s$,^1^ and applying the Farnebäck dense OF algorithm [19]. For each flow map, the average intra-pair OF magnitude was calculated.

**Fig. 3:**
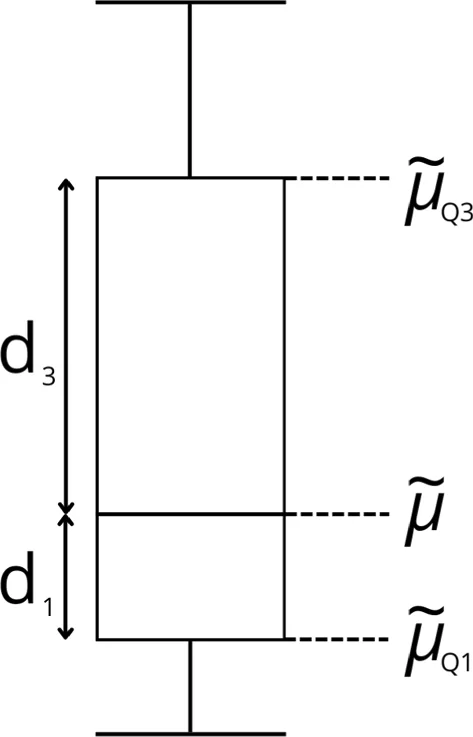
Data distribution of the optical flow (OF) magnitudes of a potential movement candidate, used for the determination of the static noise level. $\text {d}_1$ and $\text {d}_3$ represent the absolute distances between the distribution’s median, $\tilde{\mu }$, and the median of first and third quartiles, $\tilde{\mu }_{\text {Q1}}$ and $\tilde{\mu }_{\text {Q3}}$, respectively

Dense OF methods capture small motion vectors between static frame pairs caused by small pixel value oscillations that originate from the static noise of video recordings. We estimated this static noise level using a median-based characterization of the average OF magnitudes of all sampled frame pairs. The noise threshold was estimated as the minimum between the median of the third quartile, $\tilde{\mu }_{Q3}$ (cf. Fig.3), and an upper bound, $U_{ft}$.

All frame pairs with average OF magnitude below the noise threshold were considered static ("non-movement") frames. To be classified as "movement," frame pairs had to:

1. have an average intra-pair OF magnitude above the noise threshold;
2. be within two sampling periods of another frame-pair classified as "movement."

**Fig. 4:**
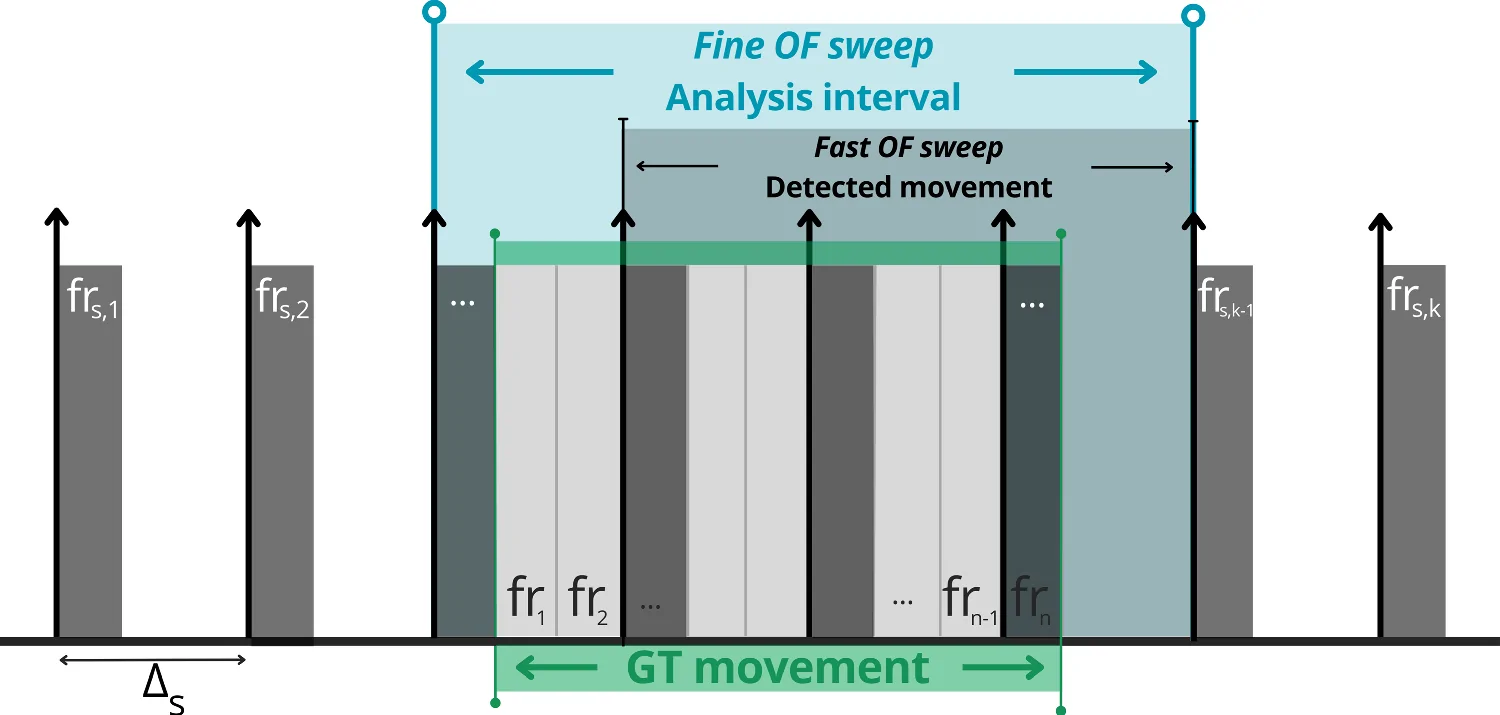
Selection of analysis interval for the *Fine OF sweep*, based on the detected movement from the *Fast OF sweep*. The analysis interval contained the static-dynamic transition frames present in the ground-truth (GT) movement annotation. $fr_{s,i}$ represents the sampled frames, at a sample period $\Delta _s$, and $fr_{j}$ the GT movement frames

As shown in Fig. 4, a secondary frame-by-frame OF sweep was implemented to detect the static-dynamic transition frames. This sweep began one sample period ($\Delta _s$) before the movement intervals detected on the first OF sweep.

#### Fine optical flow sweep

The *Fine OF sweep* analyzed the potential swallow candidates that resulted from the first sweep, to accurately pinpoint their static-dynamic transition frames, and to provide the final swallow candidate estimates. Each potential movement sequence was sampled frame-by-frame, and, as in the *Fast OF Sweep*, the Farnebäck dense OF algorithm [19] was computed between each pair of frames.

When computing dense OF fields with a shorter frame sampling interval, the average flow magnitudes, representing the overall motion, tend to be smaller. As a result, the previously used noise threshold becomes too high and is no longer suitable for distinguishing true motion from noise, requiring its recalculation. The new noise threshold, $n_{l}$, was computed using the maximum between the median, $\tilde{\mu }$, and an upper bound, $U_{ft}$, alongside the distances between $\tilde{\mu }$ and the medians of the first and third quartiles ($\tilde{\mu }_{Q1}$ and $\tilde{\mu }_{Q3}$), $d_1$ and $d_3$, respectively (cf. Fig. 3)(1).

1 $$\begin{aligned} n_{l} = {\left\{ \begin{array}{ll} \text {max}(\tilde{\mu }, U_{ft}), &  d_3 - d_1 \ge 0.5 \\ \tilde{\mu }, &  \text {otherwise} \end{array}\right. } \end{aligned}$$

As in the *Fast OF sweep*, frame sequences were classified as "movement" if their average OF magnitude exceeded the noise threshold and they occurred within one $\Delta _s$ of another "movement" frame, ensuring robustness against magnitude fluctuations. Finally, only movement segments longer than double $\Delta _s$, were considered swallow candidates.

### Post-collection synchronization of multi-swallow HRIM and VFSS continuous recordings

To achieve reliable synchronization of both signals we used a binary cross-correlation algorithm between two binary signals: $L_{b}$, derived from the real-time HRIM clinician annotations, and $C_{b}$, derived from the detected swallow candidates of the VFSS recording. The *on* values of $L_{b}$ are found between the timestamps of each annotation, while the *on* values of $C_{b}$ are found between the timestamps each found swallow candidate.

Generally, during simultaneous HRIM and VFSS, data is sampled at different frequencies. To synchronize corresponding swallows within similar time intervals, both signals were upsampled to a common frequency, $f_{up}$, chosen to be higher than the native sampling rates of both signals. This step was performed for computational convenience, as upsampling does not lead to data loss and facilitates alignment on a shared time axis.

Cross-correlation was performed using the upsampled HRIM landmark-based binary signal, $L_{up}$, as the anchor, and the upsampled candidate binary signal, $C_{up}$, as the sliding signal, each with *N* samples. Correlation values were evaluated across all positions, *k*, of the sliding binary signal ($C_{up}$), with zero padding and no circular integration. The delay was determined as the $k^{th}$ position that maximized the correlation measure, $R_{HC}$ (2).

2 $$\begin{aligned} R_{HC}[k] = {\left\{ \begin{array}{ll} \sum _{n=0}^{N+k-1} L_{up}[n] \cdot C_{up}[n-k], &  \text {if } k \ge 0 \\ \sum _{n=-k}^{N-1} L_{up}[n] \cdot C_{up}[n-k], &  \text {if } k < 0 \end{array}\right. } \end{aligned}$$

### Classification of movement segments using HRIM pressure data

To classify swallow candidates as *true* swallows we used a template-similarity analysis based on pressure distribution from HRIM-annotated swallow events.

#### Sensor selection

The analysis was based on data from two pressure sensors in the upper esophageal sphincter (UES). All included patients consistently produced a high-pressure zone in the HRIM pressure contour plot around the UES. Consequently, the two sensors, $S_1$ and $S_2$, with the highest overall average pressure values were automatically identified and selected as corresponding to the UES.

#### Template selection

After synchronization, three swallow events annotated during the HRIM acquisition that aligned with swallow candidates detected by the *Fine OF sweep* were selected as template valid. For each template valid event, the pressure segments, $p_{t_j,S_i}$ with $j \in \{1,..., \textbf{P}\}$ and $i \in \{1, 2\}$, recorded by $S_1$ and $S_2$, were extracted and pre-processed using mean removal and low-pass filtering. Each $p_{t_j,S_i}$ was characterized by three sensor-specific parameters: maximum peak-to-peak amplitude ($A_{pp-pt_{j,S_i}}$), absolute mean ($\mu _{pt_{j, S_i}}$), and standard deviation ($\sigma _{pt_{j, S_i}}$). The sensor-specific swallow template, $T_{\text {swallow}, S_i}$, was then defined by parameters $A_{pp-T_{S_i}}$, $\mu _{T_{S_i}}$, and $\sigma _{T_{S_i}}$, computed as the normalized averages of the sets of parameters $\mathbf {A_{pp-pt_{S_i}}} = \{A_{pp-pt_{1,S_i}}, A_{pp-pt_{2,S_i}},..., A_{pp-pt_{\textbf{P},S_i}}\}$, $\boldsymbol{\mu _{pt_{S_i}}} = \{\mu _{pt_{1, S_i}}, \mu _{pt_{2, S_i}},..., \mu _{pt_{\textbf{P}, S_i}}\}$, and $\boldsymbol{\sigma _{pt_{S_i}}} = \{\sigma _{pt_{1, S_i}}, \sigma _{pt_{2, S_i}},..., \sigma _{pt_{\textbf{P}, S_i}}\}$ (3).

3 $$\begin{aligned} \begin{aligned} A_{pp-T_{S_i}}&= \frac{\frac{1}{P} \sum _{p=0}^{P} A_{pp-pt_{S_i}}[p] - \min (\mathbf {A_{pp-pt_{S_i}}})}{\max (\mathbf {A_{pp-pt_{S_i}}})} \\ \mu _{T_{S_i}}&= \frac{\frac{1}{P} \sum _{p=0}^{P} |\mu _{pt_{S_i}}[p]| - \min \boldsymbol{(|\mu _{pt_{S_i}}|)}}{\max (\boldsymbol{|\mu _{pt_{S_i}}}|)} \\ \sigma _{T_{S_i}}&= \frac{\frac{1}{P} \sum _{p=0}^{P} \sigma _{pt_{S_i}}[p] - \min (\boldsymbol{\sigma _{pt_{S_i}}})}{\max (\boldsymbol{\sigma _{pt_{S_i}}})} \end{aligned} \end{aligned}$$

#### Candidate template-similarity analysis

The initial classification of swallow candidates was based on their temporal correspondence with the three HRIM live annotations selected as template valid. The swallow candidates whose timestamps overlapped with a template valid HRIM swallow event were automatically classified as *true* swallows.

The remaining swallow candidates (i.e., without overlap) were classified in two steps. First, we extracted and pre-processed the pressure segments $p_{c_m, S_i}$ for $m\! \in \! \{1,2,\ldots , \textbf{C}\}$, from the manometric data of $S_1$ and $S_2$ that aligned with the swallow candidate, using the same methods as for the template selection. Each $p_{c_m, S_i}$ was then summarized in the same three parameters as $p_{t_j,S_i}$. The normalization step was performed relative to the sets of parameters of the template valid segments, $\mathbf {A_{pp-pt_{S_i}}}$, $\boldsymbol{\mu _{pt_{S_i}}}$, and $\boldsymbol{\sigma _{pt_{S_i}}}$.

Classification used a weighted normalized loss function, $\mathcal {L}_{S_i}$, that compared the normalized parameters of each $p_{c_m,S_i}$, namely $A_{pp-c_{S_i}}$, $\mu _{c_{S_i}}$, and $\sigma _{c_{S_i}}$, with the ones from $T_{\text {swallow},S_i}$. Additionally, $\mathcal {L}_{S_i}$ attributed different weights, $w_{A_{pp}}$, $w_{\mu }$ and $w_{\sigma }$, to each term, providing lower scores to candidates that presented swallow-unfit data distributions, namely lower peak-to-peak amplitudes and standard deviations, and higher absolute mean values (4). The pressure-candidate score was a weighted average of the sensor-specific scores, $\mathcal {L}_{S_1}$ and $\mathcal {L}_{S_2}$, with corresponding weights $w_{S_1}$ and $w_{S_2}$. Finally, pressure candidates with scores above the threshold $t_{\text {score}}$ were classified as *true* swallows. Candidates scoring below this threshold were pre-eliminated and reclassified as a *true* swallow, if its absolute overall score drifted more than $t_{\text {drif}}$ from the median of all pre-eliminated candidates, $\tilde{\mu }_{\text {pre}}$, and if its overall score was greater than $\tilde{\mu }_{\text {pre}}$.

4 $$\begin{aligned} \begin{aligned} \mathcal {L}_{S_i}&= w_{A_{pp}}(A_{pp-c_{S_i}} - A_{pp-T_{S_i}}) - w_{\mu }(\mu _{c_{S_i}} - \mu _{T_{S_i}})\\&\qquad + w_{\sigma }(\sigma _{c_{S_i}} - \sigma _{T_{S_i}}) \end{aligned} \end{aligned}$$

## Results

### Framework evaluation

As previously described, the simultaneous acquisition of HRIM and VFSS in patients with HNC poses notable clinical and technical challenges. For meaningful clinical impact, the methodology must effectively replace manual processes without compromising the swallow quality diagnosis. As such, the framework was evaluated on two topics:

1. post-collection synchronization of the HRIM and VFSS;
2. and automatic swallow event detection.

#### Post-collection synchronization of HRIM and VFSS

To quantify the performance of the synchronization tool, we compared the reference delays between the start of the VFSS recording and the start of the HRIM acquisition with the estimated delays using the movement candidates of the double-sweep OF methodology. Table 1 shows the reference delays, $\Delta _{t_{\text {ref}}}$, the estimated delays, $\Delta _{t_{\text {est}}}$, and their absolute differences, $\overline{e}_{\Delta _t}$.

**Fig. 5:**
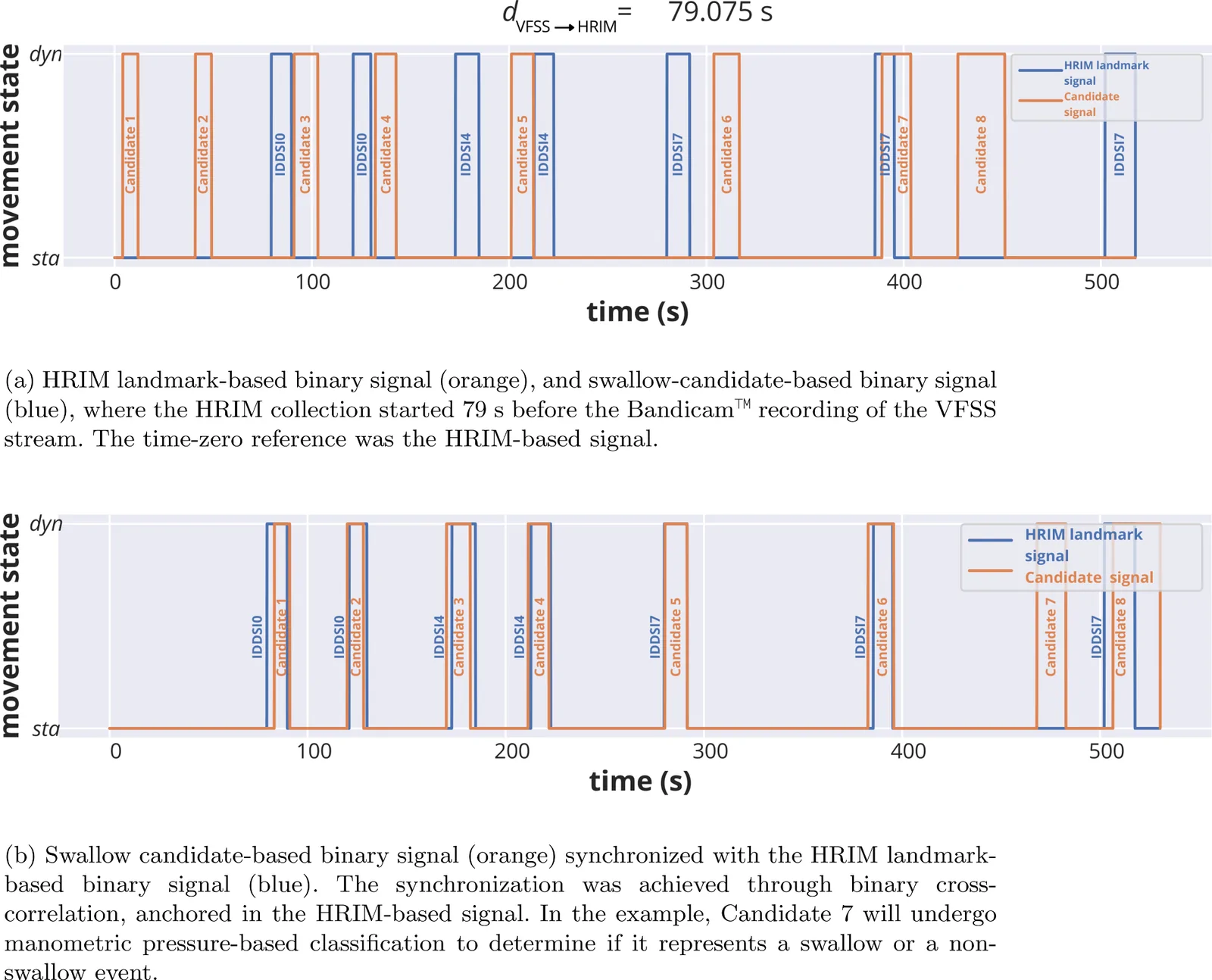
Example of post-collection synchronization of HRIM and VFSS. Visible are the original binary signals (a), and the result of the landmark-based binary cross-correlation synchronization (b)

**Table 1:** Evaluation summary of post-collection synchronization of HRIM and VFSS recordings. HRIM stream and VFSS recording were initiated at different time points during the session. The reference delays, calculated using the ground-truth annotations of both data sources, are denoted by $\Delta _{t_{\text {ref}}}$. The estimated delays, computed using the swallow candidates of the double-sweep OF, are represented by $\Delta _{t_{\text {est}}}$. The absolute difference between the reference and estimated delays is reported as $\overline{e}_{\Delta _t}$. All values are presented in seconds

ID	$\boldsymbol{\Delta _{t_{\text {ref}}}}$ (s)	$\boldsymbol{\Delta _{t_{\text {est}}}}$ (s)	$\boldsymbol{\overline{e}_{\Delta _t}}$ (s)
1	49.40	49.40	0.00
2	48.70	48.67	0.03
3	-0.60	-0.68	0.08
4	3.90	3.73	0.17
5	-8.68	-8.18	0.50
6	6.53	6.80	0.27
7	79.08	80.08	1.00
8	129.24	129.43	0.19
9	7.98	8.30	0.32
10	403.95	403.50	0.45
11	37.70	37.65	0.05
12	1.53	1.63	0.10

After synchronization, overlapping events were manually verified to ensure that aligned timestamps corresponded to the same swallow event. The synchronization tool successfully matched all swallow events across both data sources, achieving a mean absolute error of 0.26±0.28 seconds. An illustrative example of synchronization algorithm is presented in Fig. 5.

#### Automatic swallow detection in VFSS videos using HRIM pressure trends

Automatic swallow detection was evaluated using standard metrics *Precision* (P), *Recall* (R), and *F1-score* (F1) that summarize the number of *True Positive* (TP), *False Positive* (FP), and *False Negative* (FN) detections (5). As stated in Sect. 2.2, a total of 61 swallows events were used for validation.

5 $$\begin{aligned} \begin{aligned} P&= \frac{TP}{TP + FP} \\ R&= \frac{TP}{TP + FN} \\ F_1&= 2 \cdot \frac{P \cdot R}{P + R} \end{aligned} \end{aligned}$$

**Fig. 6:**
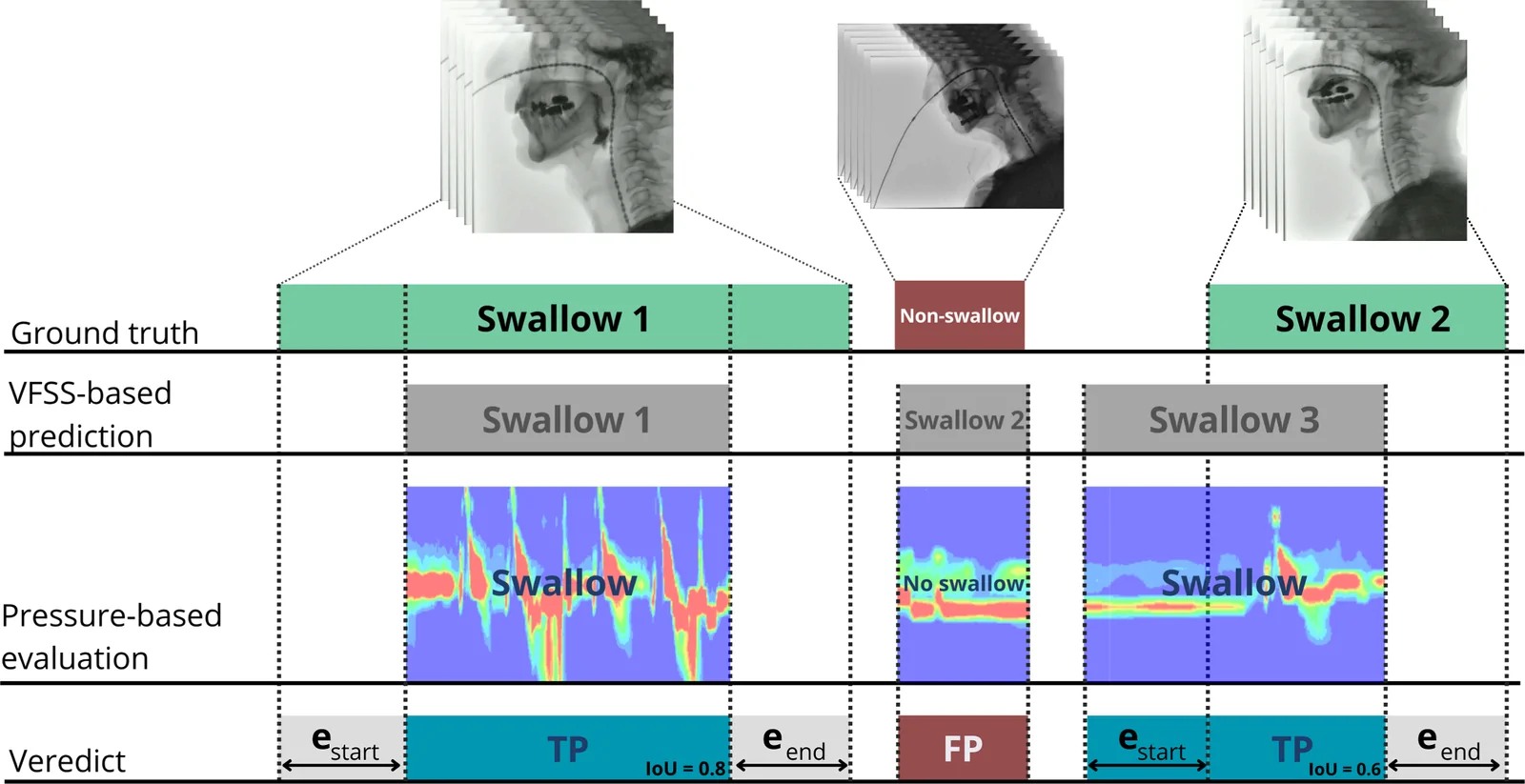
Illustration of the proposed methodologies and evaluation metrics. It is possible to observe how the pressure-based evaluation contributes to the effective elimination of *False Positive* (FP) swallow candidates proposed by the *Fine OF sweep*. Additionally, a graphical visualization of the onset and offset detection accuracy metrics, $e_{\text {start}}$ and $e_{\text {end}}$, is visible on *True Positive* (TP) swallows

As shown in Fig. 6, TP swallows segments were all movement intervals that:

1. overlapped the GT annotation with an intersection-over-union (IoU) over 50%;
2. and were classified as *true* swallows by the pressure-based evaluation.

To quantify the detection accuracy, we calculated the mean absolute difference between the start and end samples of TP swallow candidates and their corresponding GT annotations (cf. Fig. 6).

The hyperparameters ($w_{A_{pp}}$, $w_{\mu }$, $w_{\sigma }$, $w_{S_1}$, $w_{S_2}$, $t_{\text {drif}}$, and $t_{\text {score}}$) used for the pressure-based analysis were optimized using Bayesian optimization using an objective function that equally balances *Precision* and *Recall*. The proposed methodology achieved 95% *Recall*, successfully detecting and classifying 58 swallow events. The mean overall detection error associated with the TP detections was 0.44±0.96s, with a mean onset error ($\overline{e}_{\text {start}}$) of 0.44±0.67s and a mean offset error ($\overline{e}_{\text {end}}$) of 0.44±1.17s. As highlighted in Table 2, the pressure-based evaluation reduced the number of FP occurrences of the *Fine OF sweep* by 61%, contributing to an overall *Precision* of 89% and an *F1-score* of 92%.

**Table 2:** Impact of the pressure-based swallow evaluation on the elimination of *False Positive* (FP) swallow candidates proposed by the *Fine OF sweep*. $\text {FP}_{\text {noVal}}$ represents the number of FP occurrences before the pressure-based evaluation step. $\text {FP}_{\text {Val}}$ represents the final number of FP swallows inferred by the proposed methodology

Patient	1	2	3	4	5	6	7	8	9	10	11	12	Tot.
FP$_{\text {noVal}}$	3	2	1	2	1	0	4	1	1	0	0	3	**18**
FP$_{\text {Val}}$	1	0	1	0	0	0	2	0	0	0	0	3	**7**

## Discussion

We presented a novel framework to automatically detect swallows in multi-swallow simultaneous HRIM and VFSS. The framework enhances the robustness of diagnostic workflows by enabling reliable swallow detection in both synchronized and unsynchronized data. By integrating optical flow-based motion analysis in VFSS with pressure-based verification from HRIM, our methodology provides a cross-validated solution that overcomes key barriers to the adoption of simultaneous HRIM-VFSS examinations in the HNC population, such as software constraints that often lead to loss of critical swallow information. Ultimately, our framework supports a more consistent, automated swallow assessment for the HNC population.

The optimized double-sweep OF algorithm with HRIM pressure-based validation achieved an *F1-score* of 92%, with very low FP occurrences. Moreover, it achieved 95% *Recall*, outperforming similar previous approaches by Lee et al. [35] and Abdelaziz et al. [36]. Additionally, our approach does not rely on deep learning architectures, eliminating the need for large annotated datasets and reducing computational complexity. The framework’s hyperparameters were tuned to balance Recall and Precision. When optimized instead to prioritize Recall, the framework achieved 100% *Recall* and 84% *Precision*. In this configuration, however, the pressure-based validation reduced the number of FP candidates from the *Fine OF sweep* by only 33%, indicating a reduced effect on overall specificity. The FP occurrences appeared mostly due to two reasons. Firstly, the swallow candidates were selected based on an OF methodology without semantic knowledge. That means that all recorded movements, such as pre-swallow positioning and post-swallow assessments, were detected by the algorithm. Secondly, the pressure-based validation was also performed without manometric semantic knowledge of swallow events, i.e., the swallow-pressure distributions were estimated based on the pressure trends delimited by the landmarks placed by the clinician. The errors in the static-dynamic transition of TP detection mostly occur due to the annotation strategy chosen, as the irradiation moment could start and end a couple of seconds before/after the swallow event, where often no movement was noticeable and, thus, the OF analysis resulted in fields of low magnitude.

By requiring only three swallow event annotations in the HRIM stream at the time of acquisition, the framework minimizes clinician input during data collection. Clinicians therefore only need to monitor the IDDSI level and bolus consistency according to the swallowing protocol, without manually delineating every swallow event in the HRIM data. This reduction in manual effort could also decrease the number of clinicians required for simultaneous HRIM–VFSS acquisition, as a single clinician could annotate the necessary HRIM events before supervising the VFSS collection. In this way, the proposed methodology could be seamlessly integrated into the acquisition frameworks proposed by Jell et al. [47] and Geiger et al. [48], further improving the efficiency of simultaneous HRIM–VFSS data collection and analysis.

Due to the limitations of commercially available software for synchronized VFSS-HRIM acquisition, the collection protocol followed at the NKI did not synchronize both modalities at the time of collection. Consequently, retrospective analysis required temporal alignment of both data streams. To address this, we developed a synchronization tool that aligns both signals independently of their initial recording timestamps. The tool uses the timestamps of swallow candidates detected by the double-sweep OF method and cross-correlates them with the timestamps of manually annotated HRIM swallows. This fully automatic approach does not require additional clinical input, unlike synchronization based on swallowing dynamics used by Ryu et al. [46] and Yoon et al. [45], or manual timestamping. Furthermore, it ensures accurate temporal alignment of VFSS video segments with their corresponding manometric data with minimal delay errors relative to the reference delays, provided that each swallow event is fully captured in both modalities, as in our collection protocol. However, its performance depends on having sufficiently wide recording margins around each swallow event, as in protocols with narrow pre- and post-event windows the methodology does not provide sufficient precision.

Although this study was conducted on a limited cohort of 12 HNC patients, it is important to note that these patients exhibited highly heterogeneous and abnormal swallowing patterns. Achieving such high performance across this variable cohort underscores the robustness and adaptability of the proposed framework. Future studies should, however, include larger and more diverse patient groups with stratification by dysphagia severity to identify the conditions under which the framework performs optimally and to uncover potential limitations. To completely remove the need for swallow event annotation during collection, future work should focus on automatically detecting and classifying swallows in HRIM. A framework similar that proposed by Geiger et al. [41], but adapted for patients with highly altered anatomies, such as those with HNC, could be particularly beneficial. Another important future step could be the automation of swallow event analysis. In such case, a method similar to the study of Geiger et al. [48] that uses spatial information of the manometric sensors visible in the VFSS videos could be a valuable addition to streamline the analysis process.

Overall, the proposed framework showed potential to reduce clinician workload during collection and analysis of simultaneous multi-swallow HRIM-VFSS, reducing the number of required HRIM markers by 63%. However, this work represents only an initial step toward fully automated analysis of combined HRIM and VFSS data. As a proof of concept, the methodology has not yet been tested in real clinical settings, and further validation under routine clinical conditions is warranted.

**Table 3:** Patient cohort characterization. Summary of demographic and clinical characteristics of the study cohort, including gender, tumor location, treatment modality, and dysphagia severity according to the DIGEST score [49]. Abbreviations: M = male; OP = oropharynx; OC = oral cavity; NP = nasopharynx; L = larynx; S = surgery; C = chemotherapy; R = radiotherapy

ID	Age	Gender	tumor Location	Treatment	Dysphagia severity
1	46	M	OP	S, C, R	Mild
2	47	M	OP	S, C, R	Severe
3	79	M	OP	S, R	Severe
4	81	M	OC	S, R	Severe
5	71	M	OP	C, R	Severe
6	60	M	OC	S, R	Severe
7	71	M	OC	S, R	Severe
8	69	M	OC	S, R	Severe
9	61	M	NP	R	Severe
10	70	M	OP, OC	S	Severe
11	69	M	L	S	Severe
12	68	M	OP, OC	S, R	Severe

## Conclusion

In this study, we introduced a fully autonomous framework for localizing swallow events in VFSS recordings using an optimized double-sweep optical flow algorithm combined with HRIM pressure-based classification. Applied to data from 12 HNC patients, the framework achieved high accuracy (92% *F1-score,* 95% *Recall*).

The proposed framework reduces overcomes constraints of commercially available software for simultaneous VFSS-HRIM on swallowing analysis of HNC patients by requiring the annotation of only three swallow events in the HRIM feed to reliably detect the remaining swallows in the protocol, reducing clinician workload at the time of collection by 63%. By minimizing manual input, the framework also reduces the potential for human error.

The framework was validated on a small patient cohort with highly heterogeneous swallowing patterns. Nevertheless, expanding the study to a larger and more diverse population would help further characterize its performance. In future work, extending the framework toward fully automated HRIM-based swallow detection could eliminate the need for real-time clinician input and further streamline the diagnostic workflow.

Ultimately, this framework lays the groundwork for scalable, objective, and multimodal analysis of swallowing function, supporting more informed, efficient, and reproducible dysphagia diagnosis in clinical practice.

## Appendix A definitions

1. **Swallow event**: Refers to all the swallow-related activities that occur from the moment the patient is instructed to begin swallowing a single bolus until they complete any additional secondary swallows associated with that initial bolus (cf. Fig. 1).
2. **Swallow candidate**: Refers to a VFSS video segment - a set of continuous frames captured during irradiation/exposure - that has movement. This movement may represent a swallow event or any other activities unrelated to swallowing that were captured under radiation.
3. **Time zero**: Refers to the reference starting point for all timing measurements within the system. It is typically defined as the moment when a key event, such as the initiation of a swallow command or the beginning of data recording, occurs. This baseline is essential for synchronizing and accurately comparing subsequent events and measurements during the analysis.

## Footnotes

1 chosen to guarantee the validity of the Nyquist theorem.
